# *In vitro* anticancer, antioxidant and antibacterial activities of crude extract prepared from *Enteromorpha intestinalis* habited in Jeddah, Saudi Arabia

**DOI:** 10.1016/j.sjbs.2024.104026

**Published:** 2024-05-23

**Authors:** Bandar A. Al-Mur

**Affiliations:** Department of Environment, Faculty of Environmental Sciences, King Abdulaziz University, Jeddah, Saudi Arabi

**Keywords:** Cytotoxic agent – *Enteromorpha*, *Intestinalis*, GC–MS, Chemical constituents

## Abstract

The recent study purposes to evaluate the biological activities of *Enteromorpha intestinalis* gathered from Jeddah coastal area, Saudi Arabia, with respect to its phytochemical components. Our results indicated that the values ​​of moisture content, ash, total organic matter, total proteins, total lipids and total carbohydrates were 34.25 ± 5.6 %, 40.70 ± 2.3 %, 25.05 ± 1.73 %, 14.39 ± 0.8 %, 4.86 ± 6.9 % and 2.81 ± 1.4 %, respectively. The data also showed that the total phenols and flavonoids were 345.04 ± 1.50 and 320.67 ± 0.92 mg/g in the dried sample, respectively. Furthermore, four compounds were detected by HPLC at very low concentrations (quinic acid, ellagic acid, cinnamic acid, and phenanthrene) and flavonoids data confirmed the presence of apeginin, rudin, diosmin, and quercilin at high concentrations of 141.26, 11.42, 121.75, and 145.28. mg/g, respectively. The crude extract of *Enteromorpha intestinalis* exhibited cytotoxicity toward hepatocellular carcinoma cells (HepG-2 cell line) using an MTT assay with concentration range between 2 and 500 µg/mL for 48 h with IC_50 =_ 40.02 ± 3.94 µg/mL. Evidently, the *Enteromorpha intestinalis* extract had Hepatoprotective activity with IC_50_ = 447.31 ± 14.59 μg/mL. The IC_50_ activity of a crude methanol extract of *Enteromorpha intestinalis* was compared with that of an antioxidant drug (Torolox). The value (98.82 ± 1.30 μg/mL) was recorded close to Torolox (62.4 ± 0.70 μg/mL). This extract also possessed moderate antibacterial activity with inhibition zones ranging between 10 mm against *Pseudomonas aeruginosa* to 16 mm against *Escherichia coli*. **Green seaweed, along with other types of seaweed, has received significant attention in recent years. Despite their potential benefits, green seaweeds are underutilized in many parts of the world. Extensive studies on different green seaweed isolates and extracts are necessary.**

## 1 Introduction

Marine species are essential sources of natural substances with pharmacological and biological properties due to their distinct structures (Maharana et al., 2019, Pradhan et al., 2021). Because of their various biological activities, as antibacterial (Hemasudha et al., 2019), antiviral (Bouhlal et al., 2011), anticancer (Kim et al., 2011), anticoagulant (Dayong et al., 2010), antioxidant (Bleakley and Hayes, 2017), and antifouling properties (Bhadury and Wright, 2004), macroalgae are the most fascinating group of algae among them. To defend themselves from other species in their surroundings, macroalgae generate a varied range of metabolites that possess chemically active properties, such as glycerols, sterols, quinones, cyclic peptides, polysaccharides, phlorotannins, and diterpenoids. These metabolites exhibit a broad range spectrum of biological activities besides to those of carotene, glycerol, alginates, carrageenans, etc. (Rashad and El-Chaghaby, 2020). Hence, they have found usage in many medicinal settings (Pradhan et al., 2021). For nearly 240 years, European scientists have been exploring the natural history of the Red Sea. The Red Sea shoreline in Saudi Arabia was initially sampled for seaweed in the 18th century (1762) by a Danish explorer and botanist who had previously gathered seaweeds from the Jeddah Seashore.

Turn recounted the collections of algae that a British admiral named Viscount Valentia had collected from the Red Sea throughout the seventeenth century (Mohamed et al., 2006). For the remainder of the 19th century, several other laborer’s harvested marine algae originated from the Red Sea (Ibraheem et al., 2014). The calls on scientists to intensify their efforts to uncover additional physiological, environmental, and industrial benefits of Saudi Arabian macroalgae (Pradhan et al., 2021).

Taxonomically, *Enteromorpha intestinalis* belongs to the chlorophyceae family of green macroalgae. Seaweeds called intestinal herbs, or *E. intestinalis* (chlorophyte), are long (10–15 cm) and wide (1–8 mm), with a smooth, tubular structure that resembles the gut and is typically unbranched (Al-Jaber et al., 2015). This year-round growing alga is found around the world, particularly in Asian nations, where it thrives in freshwater, brackish water, and saltwater environments. Although the antioxidant and antibacterial properties of this macroalgae have already been investigated, little is known about its anticancer properties (Kosanić et al., 2015).

Although, as some previous researchers have noted, there is interest in employing *E. intestinalis* as an cytotoxic agent (Kidgell et al., 2019). Furthermore, assessments of the extracts' total phenolic component content, reducing activity, 2,2-diphenyl-1-picrylhydrasyl (DPPH) radical-scavenging capacity and inhibition of lipid peroxidation, superoxide anion scavenging activity were used to demonstrate the antioxidative potential of *Enteromorpha intestinalis* (Baek and Kim, 2019). Many processes and mechanisms, including chain initiation prevention, reductive capacity, transition metal ion catalyst adhesion and radical scavenging, have been linked to their antioxidant properties (Wassie et al., 2021).

As a result, the present investigation aimed to examine the collected algae, Enteromorpha intestinalis, especially phytochemicals, to explain their anticancer, antioxidant, and antibacterial properties. In addition, the research was expanded to characterize the principal constituents of crude extracts using gas chromatography-mass spectrometry.

## 2 Methodology

### 2.1 Algal sampling

**Fig. 1:**
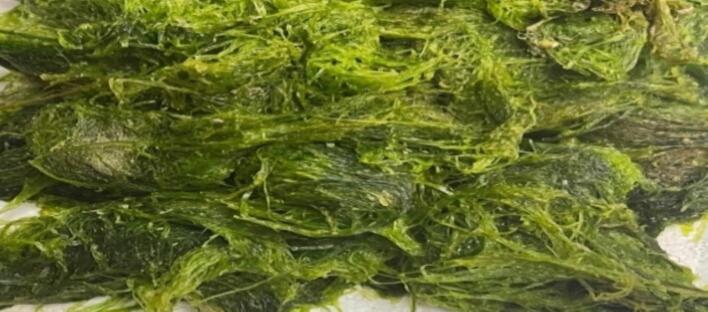
The sample of *Enteromorpha intestinalis* collected from Jeddah coastline, Red Sea, KSA*.* (For interpretation of the references to color in this figure legend, the reader is referred to the web version of this article.)

The green alga: *Enteromorpha intestinalis* (Fig. 1) was harvested from Jeddah, Saudi Arabia's Red Sea shoreline, and kept in a clean glass container. After gathering, the algae sample was cleaned with tap water to eliminate sand and debris then it was cleaned with deionized water to remove any salt residue. Finally, the sample was recognized using a standard seaweed manual (De Pádua et al., 2004). For further analyses, samples were dried at room temperature after being diced into pieces that were about 2 mm in size.

### 2.2 Phytochemical analysis

The moisture content (humidity %) and ash value were determined according to AOAC (2005). To estimate protein content, the algal sample of 100 μL was diluted 100X in PBS buffer (pH 7.4) and subsequently was subjected to sonication for a duration of 30 min. moreover 150 μL was assessed through Thermo Scientific Micro BCA Protein Assay Kit. In addition, both carbohydrates and lipids percentages were calculated by method of El-Naggar et al. (2014).

#### 2.2.1 Determination of total flavonoids and phenols

The method described by Kaur and Kapoor (2002) was used to determine total phenols. Folin-Ciocalteu reagent was used along with a 20 % w/v solution of sodium carbonate. However, the total phenolic level was evaluated and the results were expressed as the gallic acid equivalent in milligrams per gram of dry weight. On the other side, total flavonoids were estimated by Karawaya and Aboutabl (1982) procedure using quercetin solution (5–300 µg) and AICl_3_ reagent (0.1 M). Finally, the UV light was utilized to evaluate the color absorbance that generated at 445 nm for quercetin.

#### 2.2.2 Superior performance liquid chromatography

The system Thermo (Ultimate 3000) was used and comprised of; the Cromelion7 interpretation program-equipped DELL-compatible computer, the pump, and the automatic sample injector besides the DAD-3000 diode array detector with Phase C18 column reversed thermo-hypersil (RP-C18), measuring 2.5 × 30 cm. Both 0.05 % trifluoroacetic acid/acetonitrile (solvent A) and distilled water (solvent B) composed the mobile phase. All standards' and the samples' UV absorption spectra were collected in the 230–400 nm range. In addition to comparing the compounds' UV spectra to the standards', retention times can be determined, these compounds were recognized. Otherwise, Table 1 shows various process conditions and mixing ratios.

**Table 1:** The following values refer to different mixing ratios.

**Time (min)**	**Solvent (A)**	**Solvent (B)**	**Conditions**
0.000	18.0	82.0	Inj. Vol: 20 µL Flow rate: 1.0 mL/min
5.000	20.0	80.0	
12.000	40.0	60.0	
20.000	18.0	82.0	

#### 2.2.3 Extraction of bioactive ingredients from algal sample

To extract the bioactive components from the dried alga, the sample was soaked in methanol at a ratio equal to 1:10 (w/v) for 96 h at room temperature then subjected to filtration. After the solvent evaporation, the crude extract was reconstituted in 5 mL of methanol (Aldughaylibi et al., 2022).

### 2.3 Assessment of cytotoxic effects of crude extract

#### 2.3.1 Cell line propagation

The HepG-2 cells (cell line derived from human Hepatocellular carcinoma) were acquired from American Type Culture Collection and cultivated in RPMI-1640 media (purchased from Lonza, Belgium), which enhanced by the addition of 10 % inactivated fetal calf serum plus 50 µg/mL gentamycin in. The cells were cultured in a humidified atmosphere with 5 % CO_2_ at 37 °C. Three sub-culturings of the cells were carried out per week.

#### 2.3.2 Cytotoxicity evaluation utilizing viability assay

In Corning® 96-well tissue culture plates, the concentration of the tumor cell lines in the medium was 5x10^4^ cells/well for cytotoxic agent tests, and the plates were incubated for 24 h. Afterward, three duplicates of the tested compound were put to 96-well plates, resulting in twelve concentrations of the tested compound. In order to establish a control group, six vehicle controls were included for each 96-well plate. These controls consisted of either medium or a 0.5 % dimethyl sulfoxide (DMSO) solution. The method using 2,5-diphenyltetrazolium bromide (MTT) (Sigma, USA) and −(4,5-dimethylthiazol-2-yl) was utilized to quantify the overall number of viable cells after a 48-hour period of incubation. More details were explained for estimating the cytotoxic effects of crude extract in Mosmann (1983). However, the 50 % threshold for inhibition (IC50), which represents the dosage required to induce detrimental effects in 50 % of intact cells, was determined utilizing Graphpad Prism software (San Diego, USA).

#### 2.3.3 Hepatoprotective study in HepG-2 cells using MTT assay

To detect the Hepatoprotective effect of algal extract in HepG-2 cells was carried out using MTT assay (Wilson, 2000). Initially, a volume of 50 μL of MTT obtained from Sigma, USA, was introduced into each well, which already contained a volume of 100 μL of a suspension of HepG-2 cells at a concentration of 1 × 10^6^ cells/mL. Four replicates were administered for each treatment and its associated control. The plates were subjected to gentle agitation and placed in an incubator set at a temperature of 37 °C. The incubation period lasted for 4 h, during which the plates were kept in a dark environment and exposed to a 5 % CO_2_ atmosphere. The reaction was halted by the addition of 150 μL of DMSO, and a microplate reader was used to measure the samples' absorbance at 570 nm in wavelength (SunRise, Tecan, USA). In particular, one way to evaluate a cell's viability and/or quantity is by looking at its capacity to decrease MTT, which is a sign of mitochondrial activity and integrity. Hepatoprotective impact is demonstrated by measuring the cell's capacity to convert MTT into the formazan derivative following exposure to test substances. Finally, the results were displayed as a percentage of viability (PV) in the following equation: PV % =[ODc- (ODt/ODc)] x 100, where ODc represents the optical density of the control (blank) and ODt represents the optical density of the test cell sample that have been treated. The EC_50_, which represents the concentration necessary to achieve a 50 % cure rate in intact cells, was determined by analysing dose response curves for various concentrations using Graphpad Prism software (San Diego, USA).

#### 2.3.4 Assay of antioxidant activity

Ten grams of algal extract were dissolved in 100 mL of methanol to obtain concentration of 100 mg/mL. The sample was divided more to obtain final concentration of 0.2 mg algal per milliliter of methanol (Duan et al., 2006). In focus, a 96-well plate was filled with 100 μL of Hydrazyl-hydrate 2,2-diphenyl-1-picryl-hydrate (DPPH; 0.1 percent in methanol) and 100 μL of compound or sample which has been dissolved in methanol (Boly et al., 2016). After 30 min incubation period, which causes a reduction in the intensity of the DPPH color that was quantified at a wavelength of 540 nm, and the obtained values were analyzed as means. The FluoStar Omega microplate reader was utilized to record the results. Specifically, the data was analyzed utilizing Microsoft Excel, and the IC_50_ value was computed utilizing Graph Pad Prism 5 by choosing the log (inhibitor) vs. normalized response-variable slope equation for non-linear inhibitor regression. and transforming the concentrations to their log value (Chen et al., 2013).

#### 2.3.5 Assay of antibacterial activity

Seven bacterial pathogens were used to detect the antibacterial effectiveness of the algal extract dissolved in DMSO (*Escherichia coli, Pseudomonas aeruginosa, Enterococcus faecalis, Staphylococcus aureus*, *Vibrio harveyi, Klebsiella pneumoniae,* and *Vibrio fluvialis*). A volume of 100 mL of nutritional agar was inoculated with 1 mL of inoculum containing 10^8^ colony-forming units. The inoculated agar was then put into a sterile Petri dish (Amer and Ibrahim, 2019). After the agar solidification, the activity was determined utilizing the well diffusion method, which was described in Ibrahim et al. (2020).

#### 2.3.6 GC–MS (Gas chromatography–mass spectrometry) analysis

The samples' chemical analysis was examined using a direct capillary column TG–5MS (30 m × 0.25 mm × 0.25 µm film thickness) and the Trace GC1310-ISQ mass spectrometer (Thermo Scientific, USA). The startup temperature of the column oven was set at 50 °C, followed by a linear increase of 5 °C per min until reaching 230 °C. Subsequently, the temperature was maintained at 230 °C for a duration of 2 min. then, the temperature was incrementally increased at a degree of 30 °C per min until reaching the ending temperature of 290 °C, where it was maintained for a duration of 2 min. The mass spectrometer transfer line and injector temperatures were preserved at 250 °C and 260 °C, correspondingly. The carrier gas utilized in the experiment was helium with flow rate of 1 mL/min. The diluted sample, with volume of 1 µL, was injected. A solvent delay of 3 min was implemented prior to injection. EI mass-spectra were acquired in full scan mode with ionization voltages of 70 eV, including the *m*/*z* 40–1000 range. The ion source temperature was set at a constant value of 200 °C. By comparing the retention durations and mass spectra with the mass spectral databases of WILEY 09 and NIST 11, the components were identified.

## 3 Results

Herein, the alga: *Enteromorpha intestinalis* is chosen for the present study collected from Jeddah, KSA. Afterward, *E. intestinalis* was evaluated to be *in vitro* anticancer agent and how its antioxidant and antimicrobial properties are closely related in anticancer efficiency.

### 3.1 Phytochemical analysis of *Enteromorpha intestinalis* extract

Results presented in Table 2 showed the ratios of various parameters recorded in *E. intestinalis* sample. Generally, the value of moisture content was 34.25 ± 5.6 %, while ash content was higher with value of 40.70 ± 2.3 %. As well as, the percentage of total organic matter, including pigments, phenolic compounds, etc., was 25.05 ± 1.73 %. Specifically, values of total proteins, total carbohydrates and total lipids were 14.39 ± 0.8, 2.81 ± 1.4, and 4.86 ± 6.9 %, respectively. However, these values reflect the environmental conditions from which the alga was collected.

**Table 2:** Phytochemical analysis of *E. intestinalis* sample under investigation.

**Parameter**	**Value (%)**
Moisture content	34.25 ± 5.6
Ash content	40.70 ± 2.3
Total organic matter	25.05 ± 1.73
Total proteins	14.39 ± 0.8
Total lipids	4.86 ± 6.9
Total carbohydrates	2.81 ± 1.4

### 3.2 Total phenols and flavonoids of *Enteromorpha intestinalis* extract

**Fig. 2:**
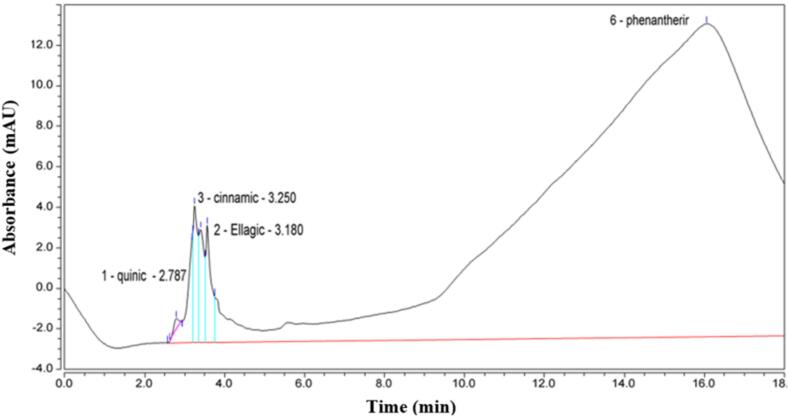
Chromatogram of phenols (types, retention times and concentrations) detected by HPLC in methanol extract of *E. intestinalis.*

**Fig. 3:**
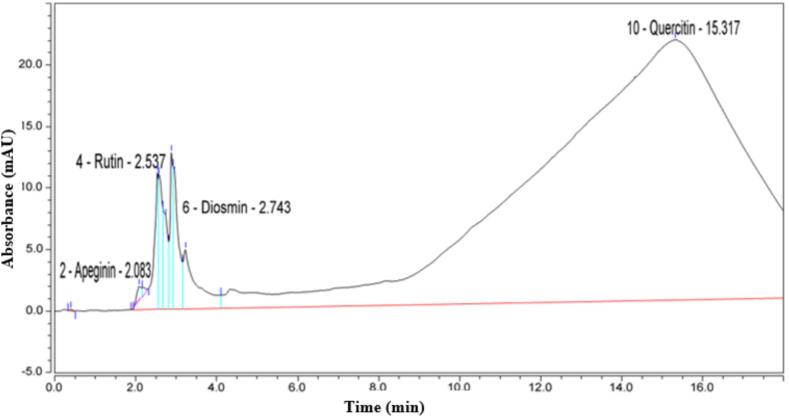
Chromatogram of flavonoids (types, retention times and concentrations) detected by HPLC in methanol extract of *E. intestinalis.*

The total phenols and flavonoids were estimated in crude extracts of *E. intestinalis* sample. Data given in Table 3 exhibited that the total phenols were 345.039 ± 1.5 mg/g in the algal dried sample. In addition, the total flavonoids in *E. intestinalis* sample were 320.670 ± 0.92 mg/g in the algal dried sample. Moreover, the HPLC chromatogram of different phenols detected in the methanol extract of *E. intestinalis,* displays their types, retention times and concentrations in both Fig. 2 and Table 4. Data revealed that four main compounds were detected but in very low concentrations. They were Quinic, Ellagic acid, Cinnamic acid, and Phenantherine with concentrations of; 0.010, 0.0006, 0.0014, and 0.0241 mg/g, respectively. Further, the HPLC chromatogram of flavonoids, detected by in methanol extract of *E. intestinalis*, presents their types, retention times and concentrations in both Fig. 3 and Table 5*.* Data confirmed the presence of Apeginin, Rudin, Diosmin, and Quercilin with rather higher concentrations of 141.263, 11.421, 121.745, and 145.281 mg/g, respectively.

**Table 3:** Analysis of total phenols and total flavonoids in *E. intestinalis* sample.

**Parameter**	**Value (mg/g)**
Total phenols	345.039 ± 1.5
Total flavonoids	320.670 ± 0.92

**Table 4:** Content of phenols including types, retention times, and concentrations, in crude extract of *E. intestinalis.*

**Peak name**	**Retention time (min)**	Area (mAU*min)	**Height (mAU)**	**Relative Area (%)**	**Relative height (%)**	**Amount (mg/g)**
Quinic	2.787	0,100	0.548	0.11	1.65	0.010
Ellagic acid	3.180	0.871	5.010	0.96	12.76	0.0006
Cinnamic acid	3.250	0.820	6.770	0.90	17.24	0.0014
Phenantherine	16.063	87.791	15.450	96.13	39.36	0.0241

**Table 5:** Content of flavonoids including types, retention times, and concentrations, in crude extract of *E. intestinalis.*

**Peak name**	**Retention time (min)**	Area (mAU*min)	**Height (mAU)**	**Relative Area (%)**	**Relative height (%)**	**Amount (mg/g)**
Apeginin	2.083	0.159	1.217	0.12	1.49	141.263
Rudin	2.537	1.534	11.152	1.20	13.70	11.421
Diosmin	2.743	1.036	7.529	0.81	9.25	121.745
Quercilin	15.317	119.439	21.161	93.32	26.00	145.281

### 3.3 Anticancer properties of *Enteromorpha intestinalis* extract

The biological properties of *E. intestinalis* have shown that its crude extract is of great value as an anticancer, antioxidant and antibacterial. First, the cytotoxicity of *E. intestinalis* extract against HepG-2 cell line was evaluated using the MTT assay under experimental conditions with a concentration range between 2 and 500 μg/mL.

**Fig. 4:**
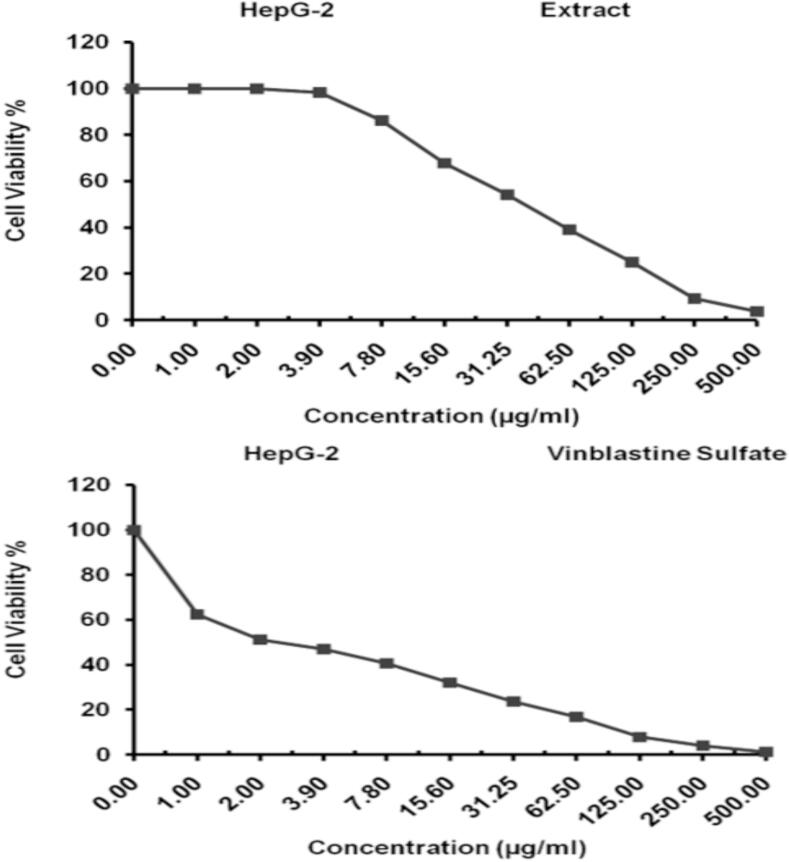
Evaluation of *E. intestinalis* extract and vinblastine sulfate in cytotoxicity expressed in cell viability % against HepG-2 cell line.

**Fig. 5:**
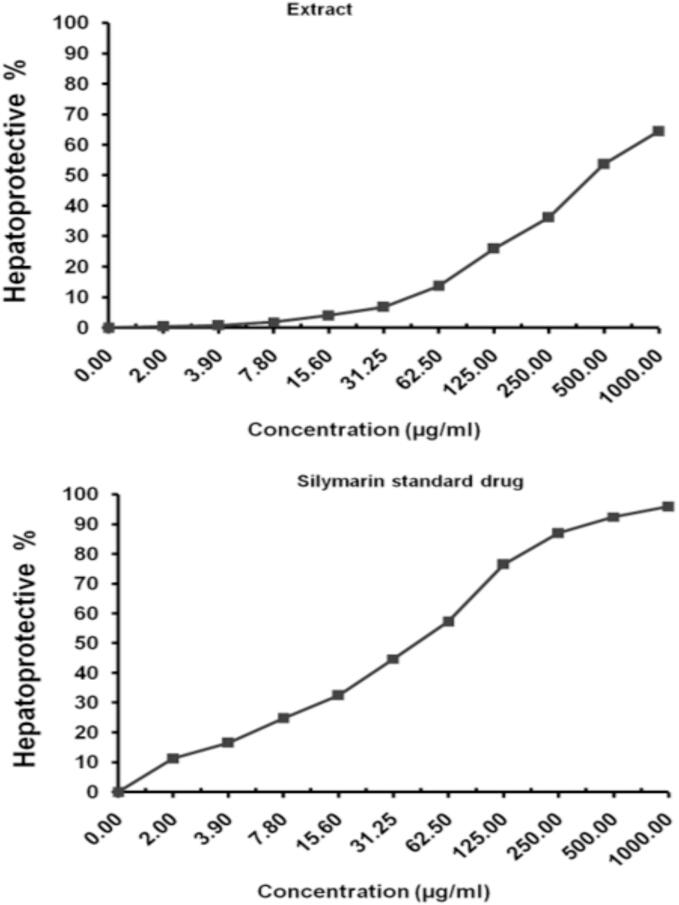
Evaluation of the *E. intestinalis* extract and Silymarin as a standard drug for hepatoprotective activity.

The data displayed in Table 6 revealed that the inhibitory effect of this extract with hepatocellular cancer cells was identified for 48 h with IC_50 =_ 40.02 ± 3.94 µg/mL. This data was more illustrated in Fig. 4. Furthermore, the cytotoxicity of vinblastine sulfate as a standard drug was estimated against HepG-2 cell line. Data in Fig. 4and Table 6 confirmed that the inhibitory action against hepatocellular cancer cells was identified for 48 h with IC_50 =_ 2.57 ± 0.19 µg/mL. Moreover, the *E. intestinalis* extract showed Hepatoprotective activity under the experimental conditions with IC_50 =_ 447.31 ± 14.59 µg/mL (Table 7 & Fig. 5). Further, the previous hepatoprotective activities were compared to Silymarin as a standard drug. However, the data in both Fig. 5 and Table 7 showed that this drug exhibited a hepatoprotective activity under the experimental conditions with IC_50_ = 44.37 ± 2.95 µg/mL.

**Table 6:** Evaluation of different concentrations from the *E. intestinalis* extract (a) and vinblastine sulfate (b) for cytotoxicity against HepG-2 cell line.

**Extract conc. (µg/mL)**	**Viability %**	**Inhibitory %**	**S.D. (±)**
500	3.87^a^ 1.23^b^	96.13^a^ 98.77^b^	0.63^a^ 0.15^b^
250	9.41^a^ 4.06^b^	90.59^a^ 95.94^b^	0.57^a^ 0.42^b^
125	25.08^a^ 7.91^b^	74.92^a^ 92.09^b^	1.73^a^ 0.57^b^
62.5	39.16^a^ 16.89^b^	60.84^a^ 83.11^b^	2.42^a^ 1.43^b^
31.25	54.23^a^ 23.68^b^	45.77^a^ 76.32^b^	1.85^a^ 2.06^b^
15.6	67.89^a^ 32.14^b^	32.11^a^ 67.86^b^	2.17^a^ 1.98^b^
7.8	86.21^a^ 40.67^b^	13.79^a^ 59.33^b^	1.43^a^ 2.05^b^
3.9	98.27^a^ 46.95^b^	1.73^a^ 53.05^b^	0.59^a^ 2.31^b^
2.0	100^a^ 51.23^b^	0.0^a^ 48.77^b^	0.0^a^ 3.49^b^
1.0	100^a^ 62.48^b^	0.0^a^ 37.52^b^	0.0^a^ 1.26^b^
0.0	100^a^ 100^b^	0.0^a^ 0.0^b^	0.0^a^ 0.0^b^

**Table 7:** Evaluation of the *E. intestinalis* extract and Silymarin as a standard drug for *in vitro* hepatoprotective activity.

**Extract conc. (µg/mL)**	**Hepatoprotective %**	**SD (±)**
1000	64.52^a^ 95.91^b^	2.06^a^ 1.03^b^
500	53.69^a^ 92.43^b^	3.17^a^ 0.91^b^
250	36.18^a^ 87.02^b^	2.84^a^ 1.44^b^
125	25.93^a^ 76.54^b^	1.35^a^ 2.08^b^
62.5	13.74^a^ 57.28^b^	0.62^a^ 1.96^b^
31.25	6.85^a^ 44.73^b^	0.43^a^ 1.29^b^
15.6	4.03^a^ 32.56^b^	0.65^a^ 1.42^b^
7.8	1.89^a^ 24.82^b^	0.17^a^ 0.74^b^
3.9	0.75^a^ 16.59^b^	0.09^a^ 0.67^b^
2.0	0.41^a^ 11.23^b^	0.13^a^ 0.25^b^
0.0	0.0^a^ 0.0^b^	0.0^a^ 0.0^b^

### 3.4 Antioxidant properties of *Enteromorpha intestinalis* extract

Secondly, to explain the anticancer of *E. intestinalis* crude extract, both its antioxidant and antibacterial properties were evaluated. Effectively, the IC50 activity of the *E. intestinalis* methanol crude extract was compared with Torolox (a standard antioxidant drugs), the former exhibited value (98.82 ± 1.3 µg/mL) nearby Torolox (62.4 ± 0.7 µg/mL). This showed that the *E. intestinalis* extract had significant antioxidant activity, making it a promising agent. These results are shown in Table 8.

**Table 8:** Antioxidant activity detected via DPPH assay referring to action of bioactive substances present in *E. intestinalis* methanol extract.

**Algal sample**	**% inhibition at 1000 μg/mL**	**% inhibition at 100 μg/mL**	**IC50 (ug/mL)**
Preliminary screening	> 50	> 50	
IC50 determination			98.82 ± 1.3
Torolox (μM)			62.4 ± 0.7

### 3.5 Antibacterial properties of *Enteromorpha intestinalis* extract

Indeed, the antibacterial properties of bioactive compounds found in the E. intestinalis crude extract were detected through the well-cut-diffusion technique. Results indicated that this extract possessed moderate antibacterial activity, through inhibition zones fluctuating from 10 mm to 16 mm (Table 9). However, *P. aeruginosa* was the most affected bacterial pathogen (16 mm), whereas E. coli was the least inhibited bacterium (10 mm). Observably, the E. intestinalis extract had no activity against S. aureus.

**Table 9:** Antibacterial activity (expressed in mm) of *E. intestinalis* extract detected via well cut-technique.

**Bacterial pathogen**	**Inhibition zone (mm)**
*S. aureus*	ND
*P. aeruginosa*	16 ± 0.33
*E. coli*	10 ± 0.57
*E. faecalis*	12 ± 0.11
*K. pneumoniae*	14 ± 0.17
*V. fluvialis*	12 ± 0.28
*V. harveyi*	14 ± 0.50

### 3.6 GC–MS analysis of *Enteromorpha intestinalis* crude extract

**Fig. 6:**
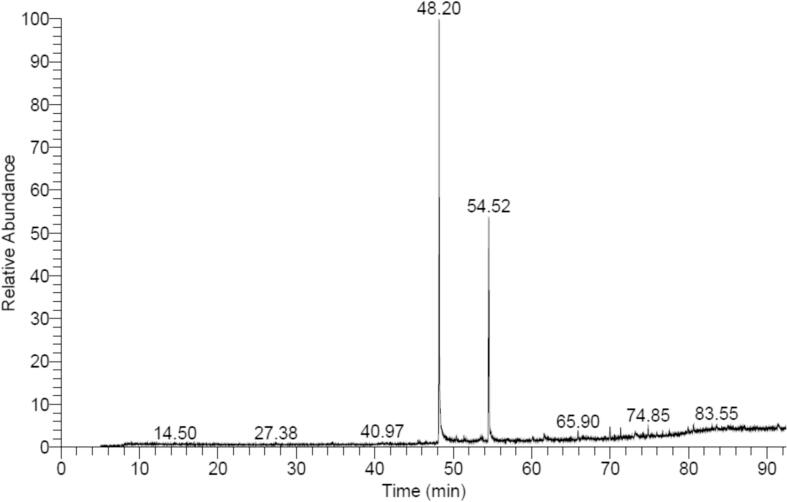
The GC–MS chromatogram of E. intestinalis methanol extract showing the retention time of the most detected compounds.

Data of GC–MS profile for the *E. intestinalis* methanol crude extract proved the occurrence of numerous active constituents, with totally 12 major compounds (Fig. 6 & Table 10). Mainly, the chemical profiles of them are: ethyl ester; methyl ester; hexadecanoic acid; octadecanoic acid; methyl ester; hexadecanoic acid, 15-methyl-, methyl ester; 3-hydroxypropyl palmitate, TMS derivative; eicosanoic acid, methyl ester; (22S)-21-Acetoxy-6à,11á-dihyd roxy-16à,17à-propylmethylened ioxypregna-1,4-diene-3,20-dio ne; glycerol monostearate, 2TMS derivative; 2-(3,4-dimethoxyphenyl)-3,5-dihydroxy-7-methoxy, and 4H-1-benzopyran-4-one; 3-cholestanol, 2-fromyl-3-benzyl; 1H-purin-6-amine, [(2-fluorophenyl)meth yl]-; and silicone oil.

**Table 10:** Most abundant chemical components detected in *E. intestinalis* methanol extract by GC–MS.

**RT (min)**	**Compound name**	**Area %**	**Probability**	**Molecular** **formula**	**Molecular** **weight (*m*/*z*)**
48. 19	Hexadecanoic acid, methyl ester	54.78	71.60	C_17_H_32_O_2_	268
50. 38	Hexadecanoic acid, ethyl ester	0.59	14.51	C_18_H_36_O_2_	284
51. 35	Hexadecanoic acid, 15-methyl-, methyl ester	0.75	28.89	C_18_H_36_O_2_	284
54. 53	Octadecanoic acid, methyl ester	32.64	70.56	C_19_H_34_O	294
61. 56	Eicosanoic acid, methyl ester	1.05	14.51	C_21_H_42_O	326
65. 90	3-Hydroxypropyl palmitate, TMS derivative	1.23	35.54	C_19_H_38_O_3_	314
69. 98	(22S)-21-Acetoxy-6à,11á-dihyd roxy-16à,17à-propylmethylened ioxypregna-1,4-diene-3,20-dio ne	1.43	41.35	C_24_H_34_O_6_	359
71. 34	Glycerol monostearate, 2TMS derivative	1.21	34.03	C_21_H_42_O_4_	358
73. 16	4H-1-Benzopyran-4-one, 2-(3,4-dimethoxyphenyl)-3,5-dihydroxy-7-methoxy	1.55	26.84	C_22_H_22_O_11_	268
74. 85	3-Cholestanol, 2-fromyl-3-benzyl	1.44	11.81	C_27_H_48_O	389
77. 54	1H-purin-6-amine, [(2-fluorophenyl)meth yl]-	0.63	31.73	C_12_H_10_FN_5_	243
79. 94	Silicone oil	0.76	24.35	[-Si(CH_3_)_2_O–]n	74.15

## 4 Discussion

Marine algae have the potential to be essential resources for the marine ecosystem because they generate the marine biological energy chain and have a wealth of bioactive substances (Morsy et al., 2020). In addition to its nutritional value, they have antibacterial, antiviral, antioxidant, anti-inflammatory, and neuroprotective properties that have been shown to enhance immune response, increase stress tolerance, and scavenge reactive oxygen species (Prabu et al., 2016).

### 4.1 Phytochemical analysis of *Enteromorpha intestinalis* extract

According to Salem et al. (2018), regarding to other algal groups, green algae (Chlorophyta) have higher carbohydrate concentration. Shankhadarwar (2015) found that E. intestinalis had a protein ratio of 8.26 mg/g and a lipid ratio of 3.3 mg/g, which were lower than the results of the current investigation. Additionally, Swathi et al. (2022) discovered that the extract of *Enteromorpha* species has a varied chemical makeup, including proteins (0.0363 g/mL) and carbohydrates (0.13 g/mL).

### 4.2 Total phenols and flavonoids of *Enteromorpha intestinalis* extract

Basically, Devi et al. (2014) reviewed that the majority of polyphenols found in the literature today are derived from seaweed or macroalgae that have been extracted from maritime environments. According to Aftab Uddin et al. (2020), the phenol content of the studied *U. intestinalis* was 149.87 mg of GAE/g that is marginally exceeding that of ethyl acetate-extracted brown seaweed. Chakraborty et al. (2013) discovered 105.97 mg of GAE/g. While, Srikonga et al. (2017) conducted that the dichloromethane extract had the highest phenolic content index of 197 mg of GAE/g per gram of extract. However, our findings showed more total phenols (345.03 mg/g) than obtained by both workers.

In contrast to our findings, no phenolic types or concentrations were obtained as obtained in recent investigations. For example, Pradhan et al. (2021) conducted *E. intestinalis* contains 60 mg of gallic acid and 625 mg of rutin equivalents per gram of extract, in terms of total phenol and flavonoids. The study by Gentscheva et al. (2022) also focused on *U. intestinalis* containing rutin and polyphenols as the highest total content. Phenolic compounds were detected by HPLC analysis in the work of Abu Samra et al. (2023) confirmed that *Enteromorpha* sp. increased its phenolic component concentration; the compounds were catechein (2.79 mg/100 g), pyrogallol (2.72 mg/100 g), vanillic (2.28 mg/100 g), and ellagic (6.15 mg/100 g). However, the last value was larger than what we found (0.0006 mg/g).

In focus, Abu Samra et al. (2023) noted that the antioxidant and anticancer effects of flavonoids and phenolic compounds in *E. intestinalis* extracts have been studied. But according to those authors, the methanolic extract of *E. intestinalis* extract produced a high phenolic concentration of 66.8 mg GAE per gram sample. Furthermore, there was a total flavonoid concentration of 49.4 mg/g in the methanol extract of *E. intestinalis*. Both values of phenols (345.0 mg/g) and flavonoids (320.6 mg/g) concentrations were lower than our detected values.

Polyphenols (particularly flavonoids) reduce oxidative damage produced by free radicals through ingesting activity. Phenolic acids have anti-inflammatory, anti-microbial, antiviral, and anticancer properties. They function as antioxidants by inhibiting the generation of free radicals (Dai and Mumper, 2010). Among the flavonoids, rutin, quercetin, naringin, kaempferol, and myricetin demonstrated the most pronounced antioxidant behavior. Quercetin exhibits superior antioxidant efficacy against free radicals that are soluble in both water and fat (Gentscheva et al., 2022). Rutin, in particular, is a phytochemical with numerous biological effects, such as analgesic, antiarthritic, neuroprotective, and antiviral activity (Zaragoza et al., 2021).

### 4.3 Anticancer properties of *Enteromorpha intestinalis* extract

In general, the anticancer properties of marine algae were documented by several authors. For instance, Lim et al. (2006) found that the MTT assay demonstrated strong anticancer activity of the *E. intestinalis* methanol extract and the variabilities of HT1080, B16/F10, and MCF7 cells reduced to 8.06, 3.62, and 10.10 %, respectively. Far from our result (IC_50 =_ 40.020 µg/mL), Paul and Kundu (2013) discovered that the methanolic extract of *E. intestinalis* was cytotoxic, showing IC50 of 309.048 μg/mL. Recently, AftabUddin et al. (2020) showed LC50 value of *U. intestinalis* extract low in cytotoxic activity.

Close to our findings, the data obtained by Abu Samra et al. (2023) demonstrated the significant anticancer activity of *E. intestinalis* methanol extract with IC_50_ (22 μg/mL) for hepatocellular carcinoma (Hep-G2) cell lines, after colon cancer (HCT-116) IC_50_ (39.21 μg/mL) and breast cancer cell lines. (MCF7) with a value of 44.0 μg/mL. Higher than our values, Saeed et al. (2020) found that the *U. Fasciata* extract has strong opposition to the PC3 and Hep-G2 cell lines (IC_50_: 12.99). and 16.75 μg/L, respectively), while *U. Lactuca* extract possessed exceptional activity towards Hela and MCF-7 cell lines (IC_50_; 10.83 and 12.43 μg/L, respectively). In particular, the high level of phenolic component in *Enteromorpha* sp., could be the reason for its antioxidant and anticancer effects (Mursi et al. (2020). Therefore, in addition to phenolic content, antioxidant qualities have been shown to explain this relationship.

### 4.4 Antioxidant properties of *Enteromorpha intestinalis* extract

Principally, antioxidant molecules are important in the management and avoidance of multiple illnesses, including atherosclerosis, cancer, chronic inflammation, and cardiovascular problems) and aging processes (Hemasudha et al., 2019). In accordance with the study's Abu Samra et al. (2023) findings, Strong correlations exist between antioxidant activity and phenolic concentration. Several workers (Saadia and Hashem, 2021, Ganesan et al., 2019) conducted that the phenolic substances concentration is commonly discovered in edible seaweeds clarified their anti-oxidative characteristics. Conversely, flavonoids are the main contributors to plant antioxidant capacity, so they are recognized to have positive health impacts (Devi et al., 2014).

Effectively, more than our IC_50_ value (98.82 µg/mL), Berber et al. (2015) displayed valuable free radical scavenging activity of all methanolic extracts with IC_50_ values fluctuating from 32.19 mg/mL to 37.57 mg/mL. Additionally, Srikonga et al. (2017) observed the highest DPPH activities in the extract of *U. intestinalis* with very low _IC50_ values as; 0.92 mg/mL and 1.50 mg/mL, correspondingly. Moreover, AftabUddin et al. (2020) confirmed that the measured IC_50_ of *U. intestinalis* to equivalent standards for ascorbic acid was 34.274 g/mL. Recently, Abu Samra et al. (2023) recorded high antioxidant activity of methanol extract of *E. intestinalis* (83.85 %) close to our finding.

### 4.5 Antibacterial properties of *Enteromorpha intestinalis* extract

Many marine algae display a varied spectrum of antifungal and antibacterial properties and have the potential to be used in such manner (Shannon and Abu-Ghannam, 2016). In terms of phenol concentration, marine algae exert an influence on bacterial cells. Such these substances damage the bacteria's membranes and cell walls (Minatel et al., 2017). Rather early, Omar et al. (2012) observed that the petroleum ether and ethyl acetate that extract of *E. prolifera* gathered from the southern shoreline of Jeddah displayed strong activity against *B. subtilis, S. aureus, K. pneumoniae, Methicillin-Resistant, S. aureus (MRSA)*, *P. aeruginosa,* and *E. coli*. Ethyl acetate extract of *E. prolifera* showed a significant maximum inhibition zone of 25 mm against MRSA more than our detection. A similar observation in our range values ​​was reported by Patra et al. (2015) where the inhibition zones were recorded between 10.0 mm and 13.3 mm. Close to our data, Srikonga et al. (2017) demonstrated the antimicrobial activity of the U. intestinalis extract, with inhibition zones fluctuating between 6.85 and 16.4 mm.

AftabUddin et al. (2020) revealed that the U. intestinalis extract presented a moderate inhibition zone (17 mm). Swathi et al. (2022) found that the *Enteromorpha* sp. extract produced larger inhibitory zones up to 18 mm towards *S. aureus* and *P. aeruginosa*, correspondingly.

### 4.6 GC–MS analysis of *Enteromorpha intestinalis* crude extract

Our result is similar to the GC–MS profiles of Kulkarni et al. (2021) demonstrated the presence of polyenes, phenols, aliphatic and phytosteroids compounds in the ethanoic extract of *U. intestinalis*. Thillairajasekar et al. (2009) also discovered that the presence of palmitic and myristic acid, stearic acid, oleic acid, linoleic acid, and lauric acid, which are known to have effective antifungal and antibacterial agents, could explain the biological activities of algal extracts. Desbois and Smith (2010) also assumed that the potent biological activities of *E. intestinalis* may came from free fatty acids (like palmitic acid, cis8-octadecanoic acid, and meristic acid) with capability to kill or prevent the growth of microorganisms. Obviously, many *Ulva* (*Enteromorpha*) extracts show antibacterial activity; numerous of these structures are identified such as fatty acids, steroids, hydroxyl unsaturated flavonoids, phenols, glycosides, terpenoids, and ghycolipids (De Alencar et al., 2016). The antibacterial activity of their organic extracts appeared to be associated with their phenolic and lipophelic concentrations (Abd El Baky et al., 2008).

## 5 Conclusion

*Enteromorpha intestinalis* is a species of green algae habited in the Red Sea in Jeddah, Saudi Arabia. Because of its abundance of bioactive substances, it is considered a promising resource. Based on its chemical profile, this investigation intends to assess its anticancer, antioxidant, and antibacterial activities. The investigation's findings indicated that the *E. intestinalis* methanol extract has an anti-carcinogenic effect, and specially the human hepatocellular carcinoma cell line was considerably affected by different concentrations of its methanol extract. Its Hepatoprotective effect in Hep-G2 cells was also demonstrated. Moreover, this crude extract recorded respectable antioxidant and antibacterial properties. In a complementary step, the GC–MS data for the *E. intestinalis* extract confirmed the presence of many bioactive constituents. They mainly were fatty acids and their derivatives. Conclusively, our results showed that *E. intestinalis* includes novel and distinct chemically active substances against the liver cancer besides it can operate as an antibacterial and antioxidant agent for future pharmaceutical applications.

## CRediT authorship contribution statement

**Bandar A. Al-Mur:** Conceptualization, Data curation, Formal analysis, Funding acquisition, Methodology, Supervision, Validation, Visualization, Writing – original draft, Writing – review & editing.

## Declaration of Competing Interest

The authors declare that they have no known competing financial interests or personal relationships that could have appeared to influence the work reported in this paper.
